# The effect of preprocessing in dynamic functional network connectivity used to classify mild traumatic brain injury

**DOI:** 10.1002/brb3.809

**Published:** 2017-09-15

**Authors:** Victor M. Vergara, Andrew R. Mayer, Eswar Damaraju, Vince D. Calhoun

**Affiliations:** ^1^ The Mind Research Network Albuquerque NM USA; ^2^ Department of Electrical and Computer Engineering University of New Mexico Albuquerque NM USA; ^3^ Departments of Neurology and Psychiatry University of New Mexico Albuquerque NM USA

**Keywords:** dynamic functional network connectivity, functional MRI, independent component analysis, traumatic brain injury

## Abstract

**Introduction:**

Dynamic functional network connectivity (dFNC), derived from magnetic resonance imaging (fMRI), is an important technique in the search for biomarkers of brain diseases such as mild traumatic brain injury (mTBI). At the individual level, mTBI can affect cognitive functions and change personality traits. Previous research aimed at detecting significant changes in the dFNC of mTBI subjects. However, one of the main concerns in dFNC analysis is the appropriateness of methods used to correct for subject movement. In this work, we focus on the effect that rearranging movement correction at different points of the processing pipeline has in dFNC analysis utilizing mTBI data.

**Methods:**

The sample cohort consists of 50 mTBI patients and matched healthy controls. A 5‐min resting‐state run was completed by each participant. Data were preprocessed using different pipeline alternatives varying with the place where motion‐related variance was removed. In all pipelines, group‐independent component analysis (gICA) followed by dFNC analysis was performed. Additional tests were performed varying the detection of temporal spikes, the number of gICA components, and the sliding‐window size. A linear support vector machine was used to test how each pipeline affects classification accuracy.

**Results:**

Results suggest that correction for motion variance before spatial smoothing, but leaving correction for spiky time courses after gICA produced the best mean classification performance. The number of gICA components and the sliding‐window size were also important in determining classification performance. Variance in spikes correction affected some pipelines more than others with fewer significant differences than the other parameters.

**Conclusion:**

The sequence of preprocessing steps motion regression, smoothing, gICA, and despiking produced data most suitable for differentiating mTBI from healthy subjects. However, the selection of optimal preprocessing parameters strongly affected the final results.

## 1 INTRODUCTION

Traumatic brain injury (TBI) has a significant impact in our society. Although traffic laws in several countries have reduced the occurrence of TBI (Redelmeier, Tibshirani, & Evans, 2003), the world health organization considers traffic accidents among the three major global concerns for disease and injury (Finfer & Cohen, 2001). In many cases TBI leads to serious short‐ and long‐term effects that impair cognitive abilities of the patient. Dangers of TBI are observed even in mild cases. Suicidality, depression, and posttraumatic stress disorder symptoms are among the deleterious effects experienced by mTBI patients (Bryan, Clemans, Hernandez, & Rudd, 2013). The wide spectrum of TBI symptoms motivates the search for new unexplored technologies that might improve the detection of mTBI.

Functional magnetic resonance imaging (fMRI) is one of the important modalities in mTBI research (Mayer, Mannell, Ling, Gasparovic, & Yeo, 2011). Several studies provide evidence that point to static functional connectivity, a data modality derived from fMRI, as promising biomarker. We call static functional connectivity the measure of coherence between brain areas evaluated over the whole period of fMRI acquisition. The potential of static functional connectivity to detect mTBI after concussions has been explored by Zhu et al. (2014). Vakhtin et al. (2013) found that the connectivity of the brain default‐mode network (DMN) (Buckner, Andrews‐Hanna, & Schacter, 2008) might be disrupted in mTBI patients. Zhou et al. (2012) found a pattern of decreased connectivity in the posterior cingulate cortex and parietal regions, but increased connectivity within the medial prefrontal cortex. Connectivity changes in the supplementary motor area and the cerebellum have been reported by Nathan et al. (2014) using a seed‐based approach. While changes in the DMN of mTBI patients were found in the study by Vakhtin et al. (2013), no significant differences, following multicomparison correction for false positives could be observed in another work by Mayer et al. (2014). Similar functional connectivity methods might deliver inconclusive results and more research is needed.

Static connectivity is a measure obtained over sufficiently long periods of time (Allen et al., 2011). Such measurements assume temporal stationarity that could result in an oversimplified analysis (Allen et al., 2012). More detail can be obtained through the dynamic functional network connectivity (dFNC) method (Allen et al., 2012; Sakoğlu et al., 2010) that attempts to analyze connectivity in relatively short periods of time. Few studies have investigated dFNC in mTBI patients, but results indicate a trend of dFNC differences in mTBI patients (Mayer et al., 2014). However, the ability to detect group differences in fMRI data might be dependent on the data preprocessing pipeline used (Damaraju, Allen, & Calhoun., 2014; Power et al., 2014; Vergara et al., 2015). In static functional network connectivity, removing motion variance early in the preprocessing pipeline leads to better detection of group differences (Vergara et al., 2015). Originally in dFNC, motion variance has been considered as a preprocessing step to be implemented after group‐independent component analysis (gICA) (Allen et al., 2012). However, studies of functional connectivity preprocessing provide evidence in favor of processing motion variance early in the pipeline (Power et al., 2014). One important concern in gICA is the data reduction step usually implemented through principal component analysis (Calhoun, Adali, Pearlson, & Pekar, 2001). Data reduction introduces nonlinear effects that have not been yet characterized in the context of gICA. Another important difference is that processing motion variance after gICA works on aggregated temporal information. The aggregated temporal information has been separated from corresponding spatial maps. In contrast, motion variance has to be processed for each voxel and may produce effects on the four fMRI dimensions. In contrast to static connectivity, dFNC is based in correlations estimated over a short period of time. For this reason, dFNC may be more sensitive to movement or spikes than the static connectivity analysis. Mentioned characteristics of data preprocessing may have an impact in dFNC analysis.

In this work we hypothesize that dFNC will be affected by the selected preprocessing. The estimation of dFNC is preceded by the use of a gICA as described by Allen et al. (2012). In our analysis, gICA decomposes the data in a set of spatial regions and corresponding time courses. As the dFNC analysis utilizes the time courses obtained from gICA, we will focus on the temporal rather than the spatial information. One of the major concerns is whether preprocessing pipelines should attempt to correct for motion variance in a voxel‐wise manner before gICA (Power et al., 2014), as opposed to performing the regression in aggregated time courses obtained after gICA (Allen et al., 2011; Mayer et al., 2014). This work explores different dFNC results obtained from different options for handling subject head motion in fMRI preprocessing pipelines.

## 2 MATERIALS AND METHODS

### 2.1 Subjects

A total of 100 subjects, 50 mTBI patients (25 females), plus 50 age (within 3 years), and gender‐matched healthy controls (HC), were included in this study. The 50 mTBI patients (mean age 27.9 ± 9.2) were recruited from local emergency rooms. Subjects classified as mTBI had a Glasgow Coma Scale (Teasdale & Jennett, 1974) between 13 and 15 at first contact with medical staff, no more than 30 min loss of consciousness (if present), and no more than 24 hr posttraumatic amnesia (if present). The inclusion criterion was based on the American Congress of Rehabilitation Medicine as described in Mayer et al. (2014). HC and mTBI subjects were excluded if there was a prior history of neurological disease, major psychiatric disturbance, and additional closed head injuries with more than 5 min of lost consciousness, additional closed head injury within the past year, learning disorder, ADHD, or a history of substance abuse/dependence including alcohol. All participants provided informed consent in accord with institutional guidelines at the University of New Mexico.

### 2.2 Imaging

All images were collected on a 3 Tesla Siemens Trio scanner located at the Mind Research Network. A 5‐min resting‐state run was completed by each participant using a single‐shot, gradient‐echo echo planar pulse sequence [TR = 2000 ms; TE = 29 ms; flip angle = 75⁰; FOV = 240 mm; matrix size = 64 × 64]. Foam padding and paper tape were used to restrict motion within the scanner. Thirty‐three contiguous, axial 4.55‐mm‐thick slices were selected to provide whole‐brain coverage (voxel size: 3.75 × 3.75 × 4.55 mm). The first five images were eliminated to account for T1 equilibrium effects. A total of 145 images were selected for further analysis. Presentation software, Neurobehavioral Systems (RRID:SCR_002521), was used for stimulus presentation and synchronization of stimuli with the MRI scanners. Subjects were instructed to stare at a foveally presented fixation cross (visual angle = 1.02⁰) for approximately 5 min and to minimize head movement.

### 2.3 Preprocessing pipelines

This work focuses on preprocessing pipelines that differ on the order of the preprocessing steps implemented. Here, the words preprocessing and pipeline are not used to designate a specific toolbox. The goal was not to test differences among known toolboxes. Instead, the focus is in the effects that preprocessing ordering has on subsequent analysis. The specific variations of the preprocessing order are described in this section as well as the definition of each pipeline.

**Figure 1:**
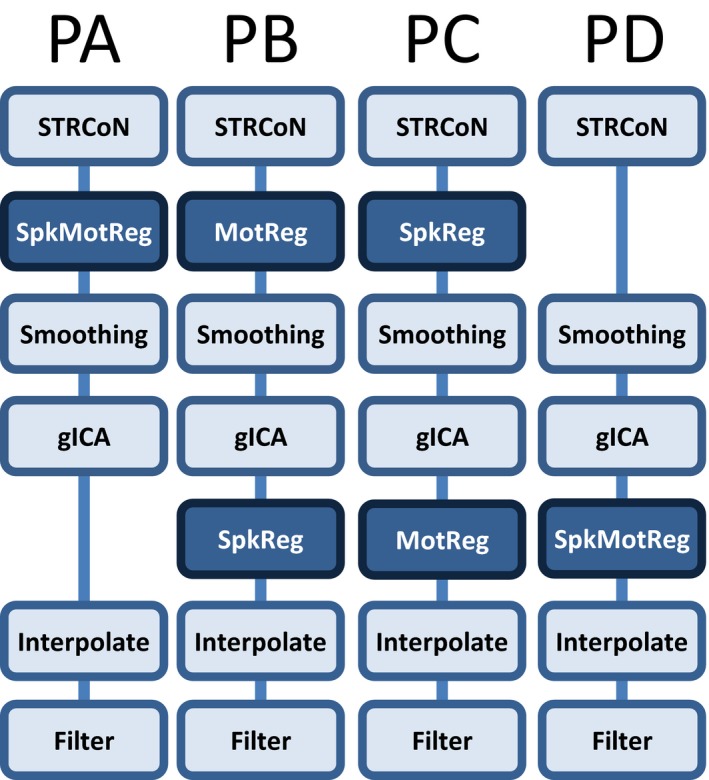
Preprocessing pipelines considered for dFNC. The main difference is the position on the pipeline where despiking (SpkReg) and motion parameters (MotReg) were regressed. SpkMotReg correspond to the combination SpkReg and MotReg. STRCoN correspond to the initial steps: slice‐timing correction, realignment, coregistration, and spatial normalization. The figure does not show variations in other parameters considered and explained in the main text

Resting‐state fMRI data were preprocessed using statistical parametric mapping version 5 (SPM 5; RRID:SCR_007037; http://www.fil.ion.ucl.ac.uk/spm) (Friston, 2003) including slice‐timing correction, realignment, coregistration, spatial normalization, and transformation to the Montreal Neurological Institute (MNI) standard space. These preprocessing steps will be designated as “STRCoN” for notation purposes. The voxel size after STRCoN was 3 × 3 × 3 millimeters. We established four different preprocessing pipelines based on the order of steps, especially the motion artifact correction. Figure 1 presents a description of each pipeline. The despiking step, designate as “SpkReg”, consisted on the orthogonalization with respect to spike regressors. Each spike is represented by an independent regressor valued 1 at the spike time point and 0 everywhere else. The DVARS method (Power, Barnes, Snyder, Schlaggar, & Petersen, 2012) was used to detect spikes and build corresponding regressors. Three different thresholds (2.5, 3.0, and 4.0 standard deviations) were considered each creating differently preprocessed datasets. There was no group difference (*p *> .50) in the number of spike regressors identified between HC and mTBI groups on any of the three thresholds. In the step designated as “MotReg”, time courses were orthogonalized with respect to i) linear, quadratic, and cubic trends; ii) the six realignment parameters; and iii) realignment parameters derivatives. In two of the pipelines, correction for spikes and motion variance are performed together using one regression analysis. This joint step is denoted as “SpkMotReg”. Smoothing and group‐independent component analysis (gICA) are performed one after the other in all four pipelines. A FWHM Gaussian kernel of 6 mm was used for the “Smoothing” step. The “gICA” step (Calhoun & Adali, 2012; Calhoun et al., 2001) was performed using GIFT (version 4.0a; RRID:SCR_001953; http://mialab.mrn.org/software/gift/) to obtain a set of functionally independent resting‐state networks (RSN) each one composed of a temporal and a spatial part (Calhoun & Adali, 2012). As this study deals with dFNC, performed analysis focuses mainly on the temporal information.

The optimal number of gICA components was determined to be 70 using a modified version of ICASSO (RRID:SCR_014981; ICASSO was included in the GIFT v4.0a package; http://mialab.mrn.org/software/gift/) (Himberg, Hyvärinen, & Esposito, 2004; Ma et al., 2011) such that the overall R‐index is close to the minimum and the index quality of at most two components falls below 0.7. The R‐index as defined in Himberg et al., (2004) is a cluster validity index (Levine & Domany, 2001) that constitutes a measure of compactness and separation of independent components. This setup was considered a good consistency trade‐off between RSN quality and clustering validity (R‐index) considering the differences among all four pipelines. However, the three numbers of components 60, 70, and 80 were considered to study the effects caused by varying this parameter. The combined steps “SpkMotReg” are applied before smoothing and gICA in pipeline A (PA), but after gICA in pipeline D (PD). In pipeline B (PB) only motion parameters “MotReg” are processed before gICA. In pipeline C (PC) only spike regression “SpkReg” is performed before smoothing and gICA. PC is similar to the pipeline commonly followed in previous mTBI FNC studies (Mayer et al., 2014) and thus represents our baseline. The order of steps in PD has been also considered in the literature (Allen et al., 2011).

The last two steps correspond to interpolation and filtering. Interpolation in this work corresponds to the replacement of spike time courses by values calculated using a cubic spline. For some pipelines spikes were also processed using a regression, which simply set spike time courses to zero. In static connectivity it is possible to simply censure spiky time courses (Vergara et al., 2015), but dFNC requires the use of interpolation to avoid discontinuities in small time windows (Allen et al., 2012). Filtering was implemented using a fifth‐order Butterworth filter with bandwidth [0.01 0.15] Hz as it has been suggested in previous dFNC literature (Allen et al., 2011, 2012).

### 2.4 Dynamic functional network connectivity

Spatial maps were z‐transformed and thresholded at |z| > 3.5 to identify brain areas of relevance in each RSN. Artifactual RSNs were detected and discarded based on their frequency content following the method proposed in Allen et al. (2011). RSNs were also manually inspected and validated by three experts who discarded RSNs if their main activation occurs in areas of white matter or cerebrospinal fluid. In addition, RSNs that could not be replicated in all four pipelines were not considered. RSN matching was performed by considering spatial correlations larger than 0.5 and by visual inspection. A total of 29 nonartifactual RSNs were selected for further analysis.

Functional relevance of each RSN was determined by assessing spatial overlap with the 90 functional regions of interest (ROI) defined by Shirer, Ryali, Rykhlevskaia, Menon, & Greicius (2012). RSN groups include subcortical (SBC), auditory (AUD), sensorimotor (SEN), cerebellum (CER), visual (VIS), salience (SAL), executive control (ECN), DMN, and language (LAN) brain regions. Thalamus and putamen constitute the SBC group. There is only one auditory RSN in the left superior temporal region. The SEN group embraces regions of the supplementary motor area and the postcentral gyrus. The VIS group includes calcarine, cuneus, occipital, and fusiform giri. Right insula and supramarginal gyrus were classified in the SAL group. Frontoparietal networks (van den Heuvel, Mandl, Kahn, Pol, & Hilleke, 2009) constitute the ECN group. The DMN is represented by angular gyrus, anterior and posterior cingulate cortexes. The LAN group consisted of left and right middle temporal gyrus.

The dFNC was estimated using the sliding time window correlation approach (Allen et al., 2012). Three different window sizes 15, 30, and 45 TRs rectangle width convolved with a Gaussian (σ = 3 TRs) were considered, each slid in steps of 1 TR. The information collected for each window consists of windowed correlations between the time courses of all RSN pairs. Obtained windows were clustered using the k‐means method with a L1‐norm distance to obtain a set of dFNC states, one for each cluster.

### 2.5 Difference between mTBI and healthy controls

Before analyzing the influence of preprocessing on classification performance it is important to gather evidence of the existence of differences between the sample groups mTBI and HC. This is important as the baseline is based on mTBI diagnosis, but not on functional connectivity. In the case of dFNC, there are a finite set of states that the brain can momentarily occupy. One simple analysis in dFNC is to tests occupancy rate differences between HC and mTBI. The occupancy rates are represented by the percentage of dFNC windows found on each state and for each subject. An unpaired *t*‐test was used to find significant differences in occupancy rates.

### 2.6 Pipeline assessment

The first step for this section was to examine the windows for each subject, identify windows belonging to the same state, and calculating a mean connectivity matrix for each state and pipeline. The frame‐wise displacement (FWD) measure of movement noise introduced by Power et al. (2012) was calculated subject wise and separated in two, one corresponding to the temporal mean taken over the three translations (TRN = mean[|△d_x_| + |△d_y_| + |△d_z_|]) and one temporal mean for the three rotations (ROT = mean[|△d_pitch_| + |△d_yaw_| + |△d_roll_|]). An additional vector containing the number of detected spikes (spk) was also utilized.

The first objective was to assess differences in functional connectivity among pipelines. A MANOVA analysis was performed within each cluster to determine if there were significant differences among pipelines. As a second‐level analysis, an ANOVA test was performed for each dFNC within the state. The analysis was repeated with the inclusion of a nuisances regression step to study the possible influence of known variability sources on functional connectivity differences among pipelines. The covariates included diagnosis, gender, age, TRN, ROT, and spk.

The second analysis seeks to find relationships between dFNC and covariates of interest (diagnosis, TRN, ROT, and spk) for each pipeline. This time the data were segregated in 16 datasets corresponding to the combinations of pipelines and states. The strength of the relationship between each dFNC and the covariates was taken as the absolute value of the regression coefficient |β| of each covariate. The linear model included vectors for diagnosis, gender, age, TRN, ROT, and spk covariates. The set of coefficients |β| were compared for each state and among pipelines utilizing ANOVA tests.

A classification procedure was performed utilizing machine learning classification and cross validation. The same covariates, except for diagnosis, were regressed out before classification and cross validation. The regression was performed separate for each pipeline and state. A linear Support Vector Machine (SVM) based on least squares with soft margin parameter C = 0.01 was utilized to classify subjects in mTBI and HC. Classification accuracy was measured using area under the curve (AUC). The overall SVM performance was assessed using leave‐one‐out cross validation (LOOCV). This way one AUC measure was obtained for each pipeline separately.

**Figure 2:**
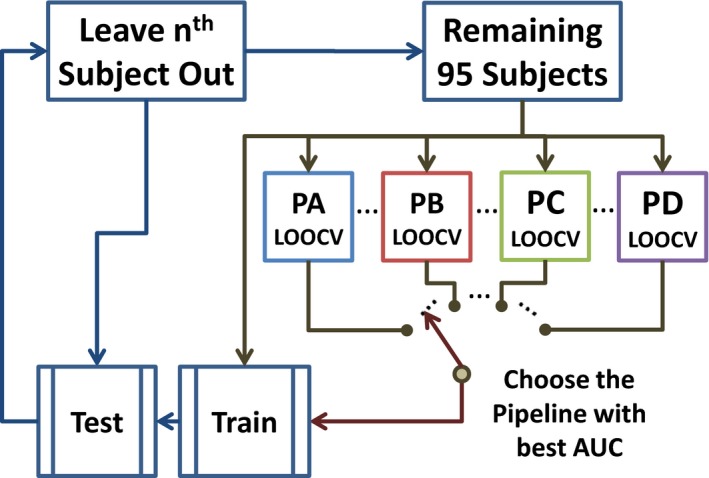
Nested LOOCV loop used to assess pipeline performances. AUC measures are obtained using an independent LOOCV for each pipeline after leaving one sample out. The SVM is then trained using data from the pipeline with largest AUC. On each inner loop there were 108 different options corresponding to the combination of four pipelines, three different spike detection thresholds, three different numbers of gICA components, and three different sliding‐window sizes. The three dots on the figure indicate the existence of parameter pipeline variations. AUCs obtained for each sample left out were stored and used for further analysis

High AUC suggests the preference of one pipeline over the others. Pipelines acts as different models of data preprocessing, but at this point model selection has not been cross validated. Problems with overfitting model selection have been described in Cawley & Talbot (2010). A nested optimization loop as displayed in Figure 2 was used to select the pipeline model and deal with model selection overfitting. The nested loop measured the AUC performance of each pipeline using a second LOOCV and the remaining samples, that is, those left after leaving the one sample out from the first LOOCV. In this second loop, the 95 remaining samples are subject to four independent LOOCV corresponding to each pipeline. The LOOCV with the highest AUC designate the pipeline with higher chance of correctly classifying the first left out sample. These four AUC are saved for further analysis. The pipeline choice is also recorded.

## 3 RESULTS

Two subjects were identified as outliers with more than 3 standard deviations on at least two frame‐wise displacement measures. These subjects and their respective matched control subjects were excluded from the analysis (Mayer et al., 2014). The final number of subjects was 96. The spatial maps of resulting RSNs can be observed in the provided Figure S1.

One hundred and eight different datasets were available after the preprocessing steps. These datasets are the results of the four different pipelines (PA, PB, PC, and PD) and the variation of parameters: three different spike detection thresholds (2.5, 3.0, and 4.0 standard deviations), three different gICA total components (60, 70, and 80), and three different dFNC sliding‐window sizes (15, 30, and 45 TRs). Each spike detection threshold, based on different standard deviations (σ*)*, detected a different ratio of spikes per subject: 2.28 for 2.5σ, 1.46 for 3.0σ, and 0.68 for 4.0σ.

**Figure 3:**
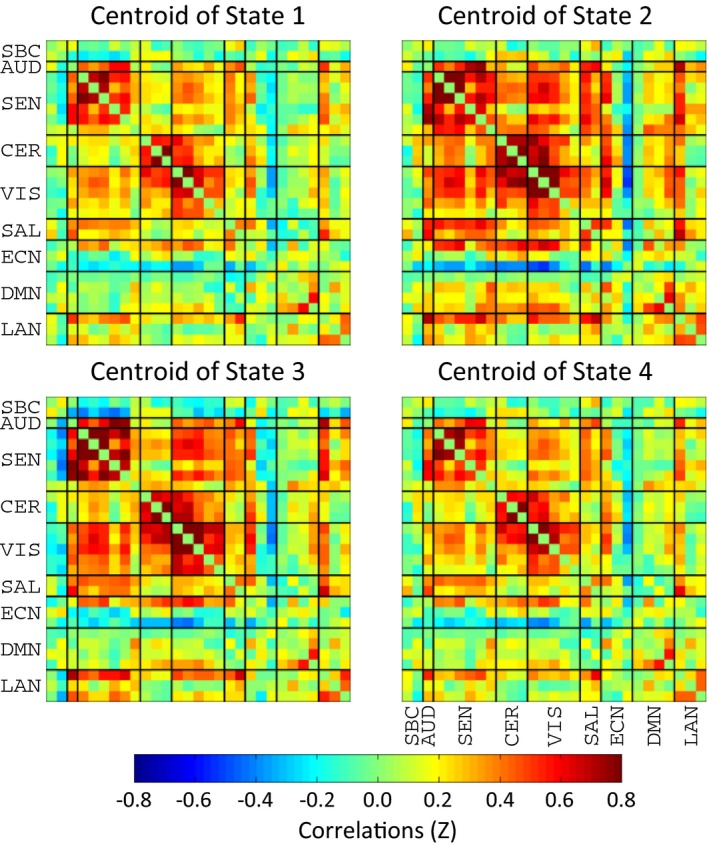
dFNC centroids obtained for each of the four clusters obtained using the k‐means algorithm. Each cluster represents a particular dFNC state. The picture displays two patterns of each strongly and weakly connected dFNC states

The application of k‐means clustering required the estimation of the number of clusters. A cluster validity index was obtained repeating the k‐means clustering and requesting a different number of clusters in the range from 2 to 8. We choose four clusters based on the elbow criteria as described in Allen et al. (2012). Centroids of obtained clusters are depicted in Figure 3. Clusters were matched among all datasets using correlation between centroids. This way, each dFNC state is represented by one corresponding cluster matched among pipelines. State 1 has the basic structure of a resting‐state matrix as it has been described before (Allen et al., 2011). State 2 is similar to State 1 with the exception of a stronger correlation between different RSN groups. State 3 is similar to State 2 except that subcortical RSNs are negatively correlated with the rest of the brain. State 1 and State 4 have few differences and are states of very low connectivity between RSN groups. The RSN groups SEN, CER, and VIS are slightly more connected in State 1 than State 4.

### 3.1 Occupancy rate results

**Figure 4:**
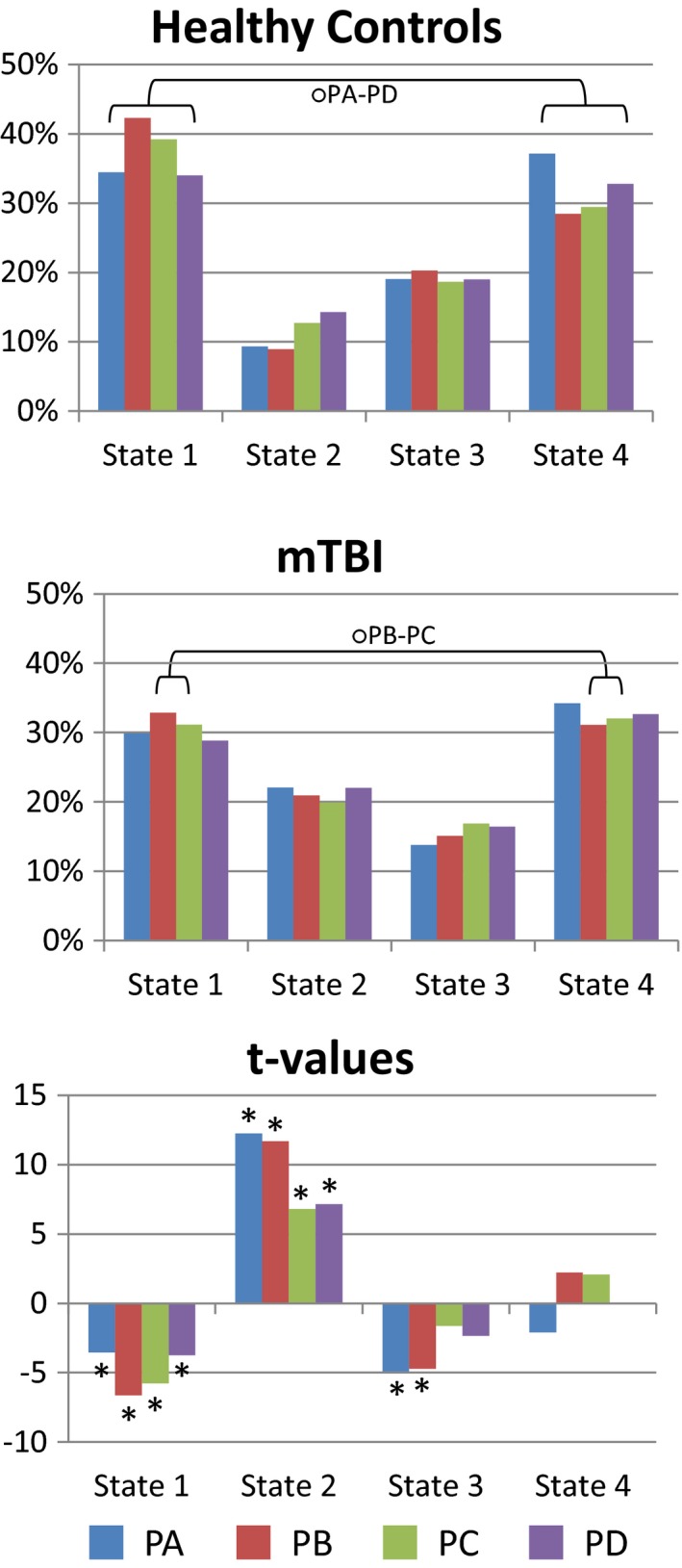
Occupancy rate results. Strongly connected states show lower occupancy rates. In the occupancy rate bars all values are significantly different (*p *< .05) except those marked with a circle (O). Significant t‐values (*p *< .05, mTBI – HC) correspond to asterisks (*)

Figure 4 shows the occupancy rate results. Data from all parameter variations were considered, but the analysis was focused on the difference between pipelines and dFNC states. More strongly connected states have lower occupancy and more weakly connected states have larger occupancy. In HC, PA and PD exhibited no significant difference in occupancy rates between State 1 and State 4, all other occupancy rates are different between dFNC states. In mTBI, occupancy rates are different between states except for pipelines PB and PC where State 1 and State 4 are not different.

The *t*‐test results show the difference between HC and mTBI samples. State 2 exhibits the largest t‐values. Significant differences (*p *< .05) are found for all pipelines in State 1 and State 2. In State 3 only pipelines PA and PB had significant differences. Although differences between State 1 and State 4 are not easily visible from the clusters in Figure 3, the difference is evident after considering the occupancy rate results where mTBI versus HC differences were detected in State 1, but not in State 4. Significant t‐values indicate that State 2 increases its occupancy in mTBI compared to HC samples, but decreases in State 1 and in State 3 only for PA and PB. This *t*‐test pattern is similar to that observed in occupancy rates.

### 3.2 Functional connectivity

**Figure 5:**
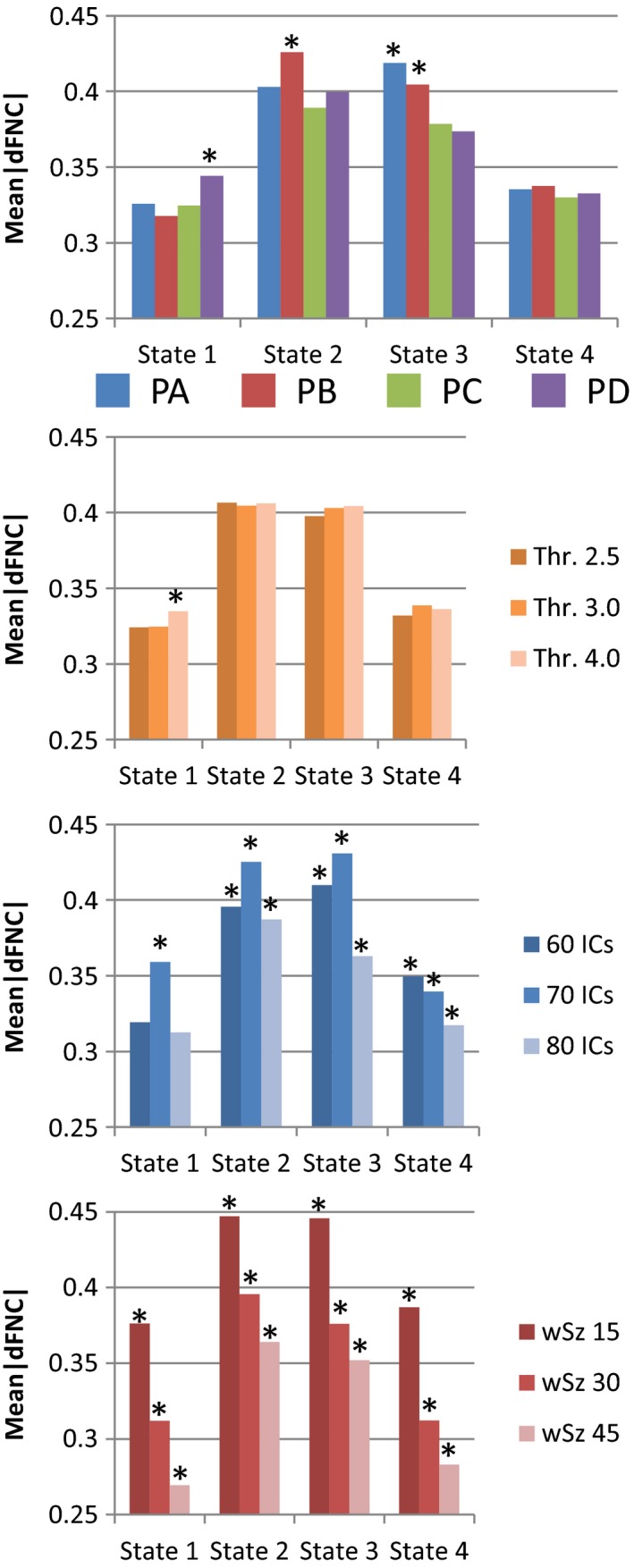
Summarized functional connectivity. The summary includes the dFNC mean of each pipeline and state. In the case of preprocessing parameters the results are organized by state. Significant results (*p *< .05) are marked with asterisk (*)

In this analysis we utilized a mean dFNC matrix per state for each subject. Differences in dFNC among the four pipelines were detected on all states after a MANOVA analysis. The largest Wilk's λ was 0.66 with the smallest χ^2^ as 2284.3 (1218 degrees of freedom). As significant differences were detected on all states, we proceeded to find those differences by second‐level ANOVA tests and summarize the results. Figure 5 displays the mean absolute value of the correlation over all dFNCs on each state. The connectivity of PD was larger in State 1 compared to the other three pipelines. State 2 exhibited no difference. In State 3, PC and PD were characterized by smaller connectivity compared to PA and PB. State 4 shows the weakest connectivity magnitude with no dFNC strength difference among pipelines. The comparison among the three spike detection thresholds indicated no significant differences among the pipelines. The number of components produced significant differences in all four states. The dFNC strength was in general larger when 70 total components were selected. Differences in sliding‐window size had the same pattern on each state with decreasing dFNC strength as the window size increases.

**Figure 6:**
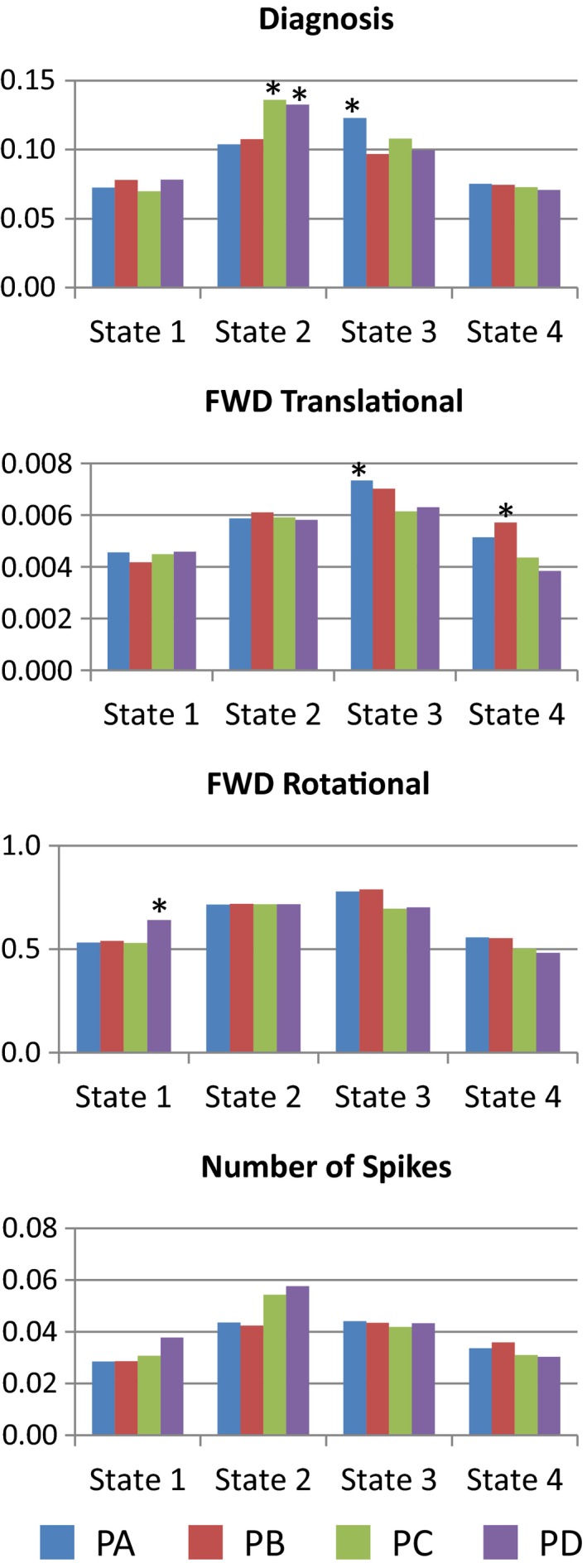
Regression coefficient strength. Results used the absolute value of the regression coefficients averaged over all 406 (29*28/2) connectivity values. The mean coefficients were compared using ANOVA tests. Asterisks indicate significantly higher (*p *< .05) coefficient magnitude within the state comparison

Figure 6 display results for the strength of regression coefficients from covariates of interest. Correlation (Corr) between diagnosis and head movement covariates was calculated to see if these two significant effects might be related, but no significant correlation was found. These measurements are as follows: Corr(dia,TRN)  = 0.16 (*p *= .12), Corr(dia,ROT)  = 0.16 (*p *= .13), Corr(dia,spk = 2.5σ)  = −0.05 (*p *= .61), Corr(dia,spk = 3.0σ)  = 0.01(*p *= .89), and Corr(dia,spk = 4.0σ)  = 0.0 (*p *= 1.0). PA showed a significantly higher strength compared to the other pipelines in State 3. However, PA also exhibit significantly higher strength with TRN in State 3. PC and PD exhibited significantly higher strength than the other pipelines. However, PD was significantly affected by the number of spikes in State 2. PA and PB were more affected by the TRN covariate, and PD by ROT and spikes. PC was the only pipeline that lacked significant results with nuisance covariates in Figure 6.

**Figure 7:**
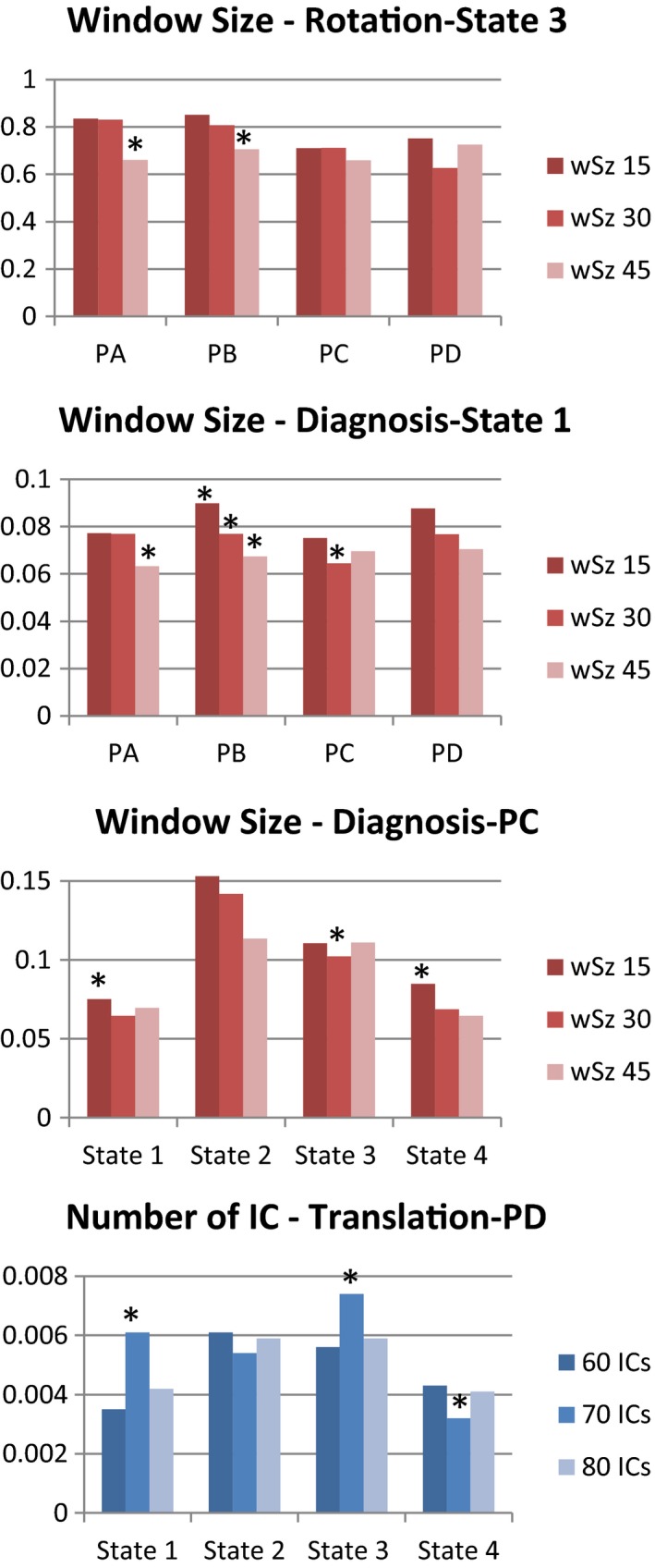
Significant differences in regression coefficient strength tested on each parameter. Differences were tested by setting either the state (varying pipeline) or the pipeline (varying the state). Window size was the most influential parameter. The number of independent components also produced a noticeable difference

Further analysis was performed on the different parameters by restricting the data to a specific state or pipeline. The significant results are presented in Figure 7. The parameter producing more differences across the different tests was the sliding‐window size. The observed trends were decrements of the mean dFNC strength (see Figure 5) and the diagnosis coefficient magnitude (see Figure 7) linked to increments of window size. In addition, the number components affected the TRN covariant for the PD pipeline. However, PD did not show this trend in Figure 6.

### 3.3 Classification results

The classification results for the separate single LOOCV pipelines were as follows: PA(63%), PB(71%), PC(66%), and PD(66%). These results were obtained by applying an independent LOOCV for each pipeline resulting in four values where variability cannot be studied. At first it seems that PB achieves the highest classification, but this result must be cross validated to study the stability of picking PB.

**Figure 8:**
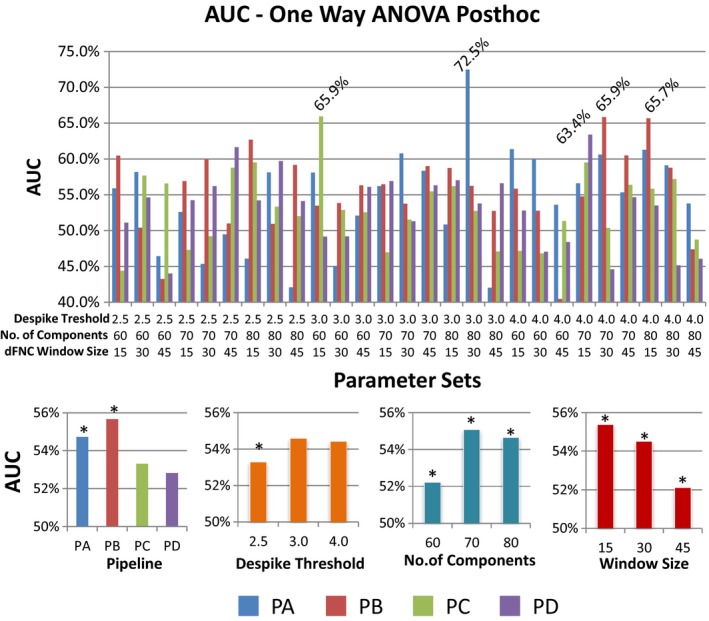
Classification performance results. Data obtained from the nested LOOCV scheme depicted in Figure 2 allow the estimation of classification performance variability by subject. An AUC value was calculated for each subject left out. This figure shows the mean AUC values averaged on the subject dimension. After a bootstrapped null model only five AUC were determined to be significantly larger than chance. These AUC values were labeled in this figure. Four extra ANOVA tests were used to study the effect that each variation of pipeline and parameters has on the mean AUC. The significant differences (*p *< .05) are indicated by an asterisk (*)

At this point there are dFNC features available from 108 different datasets representing different pipelines and parameter settings. Although one LOOCV‐SVM can be run for each dataset, results obtained this way are not suitable for comparing the different datasets. The nested double LOOCV in Figure 2 was designed to measure these characteristics as on each outermost LOOCV loop the datasets enter a contest and the best is chosen to then classify. The number of times each dataset was selected as the best to use for training was counted for each of the 96 subjects. As a result the dataset with PA as pipeline, a threshold of 3.0, a number of components of 80, and a sliding‐window size of 45 TRs was chosen 93 times. The dataset with PB as pipeline, a threshold of 4.0, a number of components of 80, and a sliding‐window size of 15 TRs was chosen for the remaining three iterations. No other dataset was chosen as the best on any iteration. The final classification had an AUC of 73%. This result indicates high model stability for the dataset chosen 93 times. After this analysis, a total of 96 AUC measurements were available for each of the 108 datasets given the way the nested LOOCV in Figure 2 works. Figure 8 displays plots of mean AUC obtained from the LOOCV implementation shown in Figure 2. After bootstrapping the 10368 (108 datasets X 96 subjects/LOOCV loops) AUC values to estimate a null model we found five significant AUCs. Each pipeline has at least one AUC result higher than chance, being PD the smallest one with 63.4%. Coincidentally the same dataset that was chosen 93 times by the nested LOOCV also exhibited the highest mean AUC of 72.5%. Further analyses were performed to investigate differences solely for pipeline and parameters. This time PB showed the highest mean AUC after considering only pipeline differences. Although a specific combination of parameters using PA resulted in the highest AUC, PB achieved higher performance after averaging AUCs from different parameter combinations. PC and PD had similar performance, but smaller than the other two pipelines. Results for despike threshold indicate that 2.5σ, the smallest threshold, also resulted in lower AUC. The AUC was significantly higher when using 70 total components. Finally, the AUC significantly decreases as the sliding‐window size increases. The AUC trends for number of components and sliding‐window size are similar to those found in Figure 5, but the despike threshold followed a different pattern.

## 4 DISCUSSION

This work tested different preprocessing pipelines observing the effect produced in dFNC results. In addition, pipelines were tested using differences among important parameters of the dFNC analysis. Just as it was observed for static connectivity (Vergara et al., 2015) preprocessing impacts the results obtained in dFNC. To the best of our knowledge, this is the first time preprocessing have been tested in dFNC analysis. This work provides further evidence that pipelines where motion variance is processed before gICA tend to deliver stronger group differences. These results agree with suggestions for ROIs preprocessing where motion variance is removed before smoothing and ROI processing (Power et al., 2012, 2014). Although gICA and ROI are different techniques for time‐series estimation, results are in favor of following the recommendation for ROI where realignment parameters are regressed early in the preprocessing.

One of the first assumptions in this work is that dFNC differs between HC and mTBI subjects. Static FNC difference has been utilized in the past to classify these two sample groups indicating that connectivity differences actually exist (Vergara, Mayer, Damaraju, Kiehl, & Calhoun, 2017). In the present analysis, occupancy rate differences provided evidence that dFNC data also differs between these two sample groups. In addition to the numerical observations presented here, there are several studies that support the assumption of functional connectivity differences. We can find in the literature examples where mTBI subjects exhibit increased functional connectivity when compared to controls. Sours et al. (2013) reported increased connectivity between salience and task‐positive networks. The connectivity between cerebellum and the SMA has been found to be stronger in mTBI patients (Nathan et al., 2014; Vergara et al., 2015). The evidence points to a pattern of increased connectivity involving salience, sensorial, auditory, and visual areas (Mayer et al., 2011, 2014). Given that State 2 represents a state with strong connectivity across the brain, increased occupancy rates for this state could explain why higher connectivity is observed in static functional connectivity. Another important characteristic is that larger occupancy rates for the state represented by State 2 decreases the occupancy in all the other states. Decreased occupancy for State 3, where subcortical networks had a more negative dFNC value, might affect overall subcortical connectivity. This provide evidence for a thalamic abnormality based on increased functional connectivity as it has been previously reported in the literature (Sours, George, Zhuo, Roys, & Gullapalli, 2015; Tang et al., 2011). This is reflected in State 2 where the thalamus has positive correlations with other RSNs in the default mode and the cerebellum groups. This correlation enhancement can be explained by a compensatory mechanism for detrimental sensorial symptoms in mTBI patients (Sours et al., 2015). Structural corticothalamic abnormalities of white matter in TBI patients are linked to more serious symptoms including posttraumatic stress disorder (Yeh et al., 2014). Obtained results cannot directly explain decreased connectivity linked to the DMN reported by previous studies (Bonnelle et al., 2011; Sharp, Scott, & Leech, 2014; Sharp et al., 2011). Our analysis is in favor of a stronger anticorrelation relationship between DMN and other cortical regions. If the DMN interference hypothesis is correct (Sonuga‐Barke & Castellanos, 2007), this connectivity enhancement might indicate a rupture of the balance between DMN and task‐positive networks affecting goal‐directed attention. Based on this discussion, we assume that diagnosis information is effectively related to dFNC contrast between mTBI and HC samples. Furthermore, our focus is to identify the preprocessing pipeline sensitive to this dFNC contrast and less sensitive to parameter selection and nuisance signals.

The results for the dFNC strength in Figure 5 suggest that residual head movement variance may have a significant effect on some dFNC state. This can be deducted from the fact that preprocessing motion parameters before gICA resulted in a trend of higher absolute value of dFNC strength for PA and PB in State 2 and State 3. These two states also exhibit higher occupancy rates and occupancy rate differences between mTBI and HC. Together, these observations suggest that PA and PB are appropriate pipelines for the detection of increased static connectivity in mTBI samples (Sours et al., 2013; Vergara, Mayer, Damaraju, Hutchison, & Calhoun, 2017; Vergara et al., 2015) previously mentioned. However, this panorama is not fully clear for the results in Figure 6. The relationship between dFNC and diagnosis was higher for PC and PD in State 2, but higher for PA in State 3. Although PC and PD exhibited increased relationship with diagnosis in State 2, the occupancy rate is smaller for this state which explains why this increased sensitivity with diagnosis is not similarly observed in static connectivity (Vergara, Mayer, Damaraju, Hutchison, et al., 2017). These results suggest that the difference between static and dynamic connectivity is rooted on the difference in occupancy rate instead of connectivity strength. In contrast, the results from classification performance suggest that PC and PD contain less information useful to distinguish HC from mTBI. The AUC results in Figures 8 and 4 agree that regressing motion parameters before gICA, as was performed in pipelines PA and PB, creates better sensitivity to diagnosis, but without pointing to a specific state. Classification results suggest that a particular parameter combination in PA produced a very stable model selected 96.8% of the times by the nested LOOCV. Although the maximum AUC result was obtained in PA for a specific combination of parameters, PB had a higher mean AUC than PA after averaging results from considered parameter combinations. In general, PB allowed the best differentiation between HC and mTBI in case variations of preprocessing parameters cannot be cross validated as is the case of most studies and applications.

Differences in preprocessing parameters exhibited different trends. The threshold used for spike detection was the parameter that resulted in the fewest number of observed effects. The most notable observation was a reduction in the AUC when using 2.5σ compared to the other two thresholds. This difference does not seem to be related to the difference in number of spikes between HC and mTBI subjects as the correlation with diagnosis was not significant and lower than 0.05. The difference could be explained by particular differences in the spike magnitude and their effect on connectivity (Damaraju et al., 2014). However, results in Figure 5 indicate this effect on connectivity is not a major source of differences and was observed in only one state. It is possible that the SVM was better at detecting the difference caused by spikes than the other analyses considered in this work. As a parameter, the total number of independent components had a consistent pattern through all analyses. Results for 70 components exhibited higher mean connectivity and higher mean classification performance than the other two options. These results indicate an optimal number of gICA components as 70, such as it was initially determined using ICASSO. There was only one unfavorable relationship with translational head motion affecting PD, as displayed in Figure 7. However, the effect was not detected on the other three pipelines. The pattern for sliding‐window sizes was also very consistent. Smaller sizes produced increases in several measures including mean dFNC, diagnosis regression coefficient, and mean classification performance. Windows with a shorter temporal span might allow for the detection of differences in temporal variations between mTBI and HC. In summary, evidence indicates that an optimal number of gICA component estimation and shorter sliding‐window sizes allow for a higher sensitivity of group differences.

Besides the different parameters tested, the pipelines considered in this work varied only on the position that spikes (SpkReg) and residual motion variance (MotReg) preprocessing steps occupy on each pipeline. Specifically, the order of steps varied according to the position of SpkReg and MotReg before or after smoothing and gICA. The implementation of SpkReg utilized was to censor spiky time courses. We proceeded with spike censoring following previous suggestion in the literature (Grouiller et al., 2011; Lemieux, Salek‐Haddadi, Lund, Laufs, & Carmichael, 2007). In contrast, other studies suggest to remove spike time points by replacing the spike point with an interpolated value extracted from surrounding (no spike) times values (Allen et al., 2011). This second approach seems necessary for dFNC analysis (Allen et al., 2012). The effect of the SpkReg step in PB and PD might have been redundant as applied interpolation step obliterated spike censoring. Nevertheless, testing SpkReg before smoothing in PA and PC allowed us to observe how it would affect the final results. The main difference for PA and PC is that spikes censoring was performed in a voxel‐wise manner thus removing larger quantities of information than in PB and PD. The best classification result was observed within the set of PA datasets. In general, PA and PB achieved the best classification performances with PB being best in the mean AUC, but PC and PD gave a similar and smaller performance. This result agrees with previous observation in static functional connectivity indicating that PA achieves the highest classification performance, followed by PB, for the same dataset utilized here (Vergara et al., 2015). Both PA and PB are pipelines with MotReg performed before smoothing and gICA (see Figure 1) consistently suggesting that motion variance should be dealt with before gICA.

One limitation in our study was the scan duration of 5 min. Several studies utilize larger scan time which may provide a better chance of observing dFNC patterns per subject (Hutchison et al., 2013). However, evidence suggests that 5 min is enough to acquire a stable connectivity signal (van Dijk et al., 2010) and is thought as minimum necessary (Allen et al., 2012). Another limitation is that we preferred a spike preprocessing method where spike repressors can be included with FWD repressors in one linear model. However, other methods to handle spikes information may have different effects.

In conclusion, the choice of preprocessing steps order significantly affects final results. Removing motion variance before smoothing and gICA, but handling spikes information after gICA as in PB might be the best bet to provide higher sensitivity to diagnosis contrasts in dFNC. This sensitivity is highly dependent on preprocessing parameters selected with a trend of better classification performance for smaller sliding‐window sizes and an optimal number of gICA‐independent components.

## CONFLICT OF INTEREST

None declared.
