# Supplementary material for: The effect of preprocessing in dynamic functional network connectivity used to classify mild traumatic brain injury
Source: Brain Behav. 2017 Sep 15;7(10):e00809. doi: 10.1002/brb3.809 (PMC5651393; doi:10.1002/brb3.809)
Supplement: Supplementary file 1 [file BRB3-7-e00809-s001.pptx]

## Slide 1
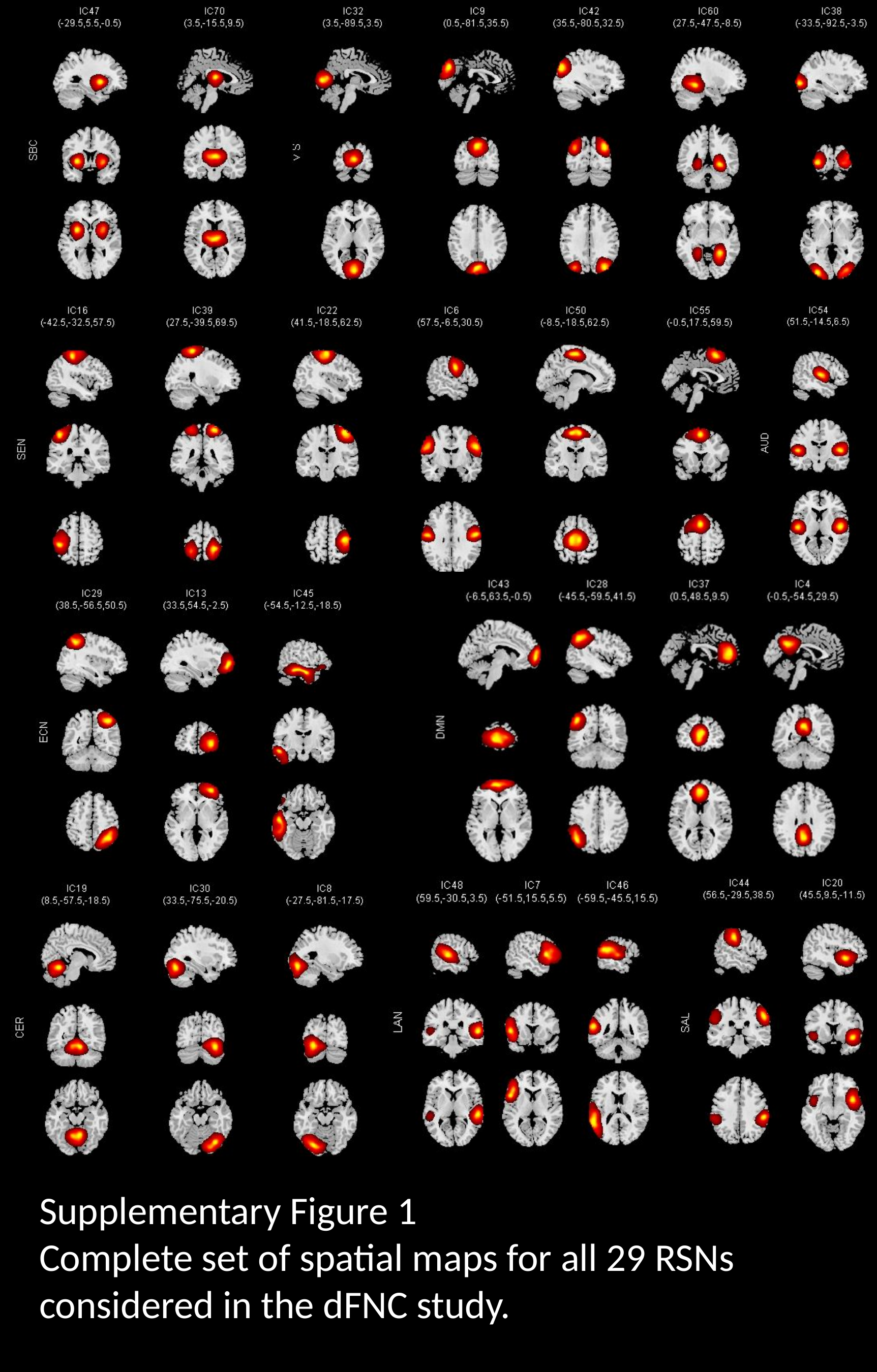

Supplementary Figure 1
Complete set of spatial maps for all 29 RSNs considered in the dFNC study.
